# The evolution of ovarian somatic cells characterized by transcriptome and chromatin accessibility across rodents, monkeys, and humans

**DOI:** 10.1093/lifemedi/lnae028

**Published:** 2024-07-31

**Authors:** Qiancheng Zhang, Fengyuan Sun, Ruifeng Zhang, Donghong Zhao, Ran Zhu, Xin Cheng, Xin Long, Xinling Hou, Rui Yan, Yu Cao, Fan Guo, Long Yan, Yuqiong Hu

**Affiliations:** State Key Laboratory of Stem Cell and Reproductive Biology, Institute of Zoology, University of Chinese Academy of Sciences, Chinese Academy of Sciences, Beijing 100101, China; Institute for Stem Cell and Regeneration, Chinese Academy of Sciences, Beijing 100101, China; Beijing Institute for Stem Cell and Regenerative Medicine, Beijing 100101, China; Key Laboratory of Organ Regeneration and Reconstruction, Chinese Academy of Sciences, Beijing 100101, China; State Key Laboratory of Stem Cell and Reproductive Biology, Institute of Zoology, University of Chinese Academy of Sciences, Chinese Academy of Sciences, Beijing 100101, China; Institute for Stem Cell and Regeneration, Chinese Academy of Sciences, Beijing 100101, China; Beijing Institute for Stem Cell and Regenerative Medicine, Beijing 100101, China; Key Laboratory of Organ Regeneration and Reconstruction, Chinese Academy of Sciences, Beijing 100101, China; State Key Laboratory of Stem Cell and Reproductive Biology, Institute of Zoology, University of Chinese Academy of Sciences, Chinese Academy of Sciences, Beijing 100101, China; Institute for Stem Cell and Regeneration, Chinese Academy of Sciences, Beijing 100101, China; Beijing Institute for Stem Cell and Regenerative Medicine, Beijing 100101, China; Key Laboratory of Organ Regeneration and Reconstruction, Chinese Academy of Sciences, Beijing 100101, China; State Key Laboratory of Stem Cell and Reproductive Biology, Institute of Zoology, University of Chinese Academy of Sciences, Chinese Academy of Sciences, Beijing 100101, China; Institute for Stem Cell and Regeneration, Chinese Academy of Sciences, Beijing 100101, China; Beijing Institute for Stem Cell and Regenerative Medicine, Beijing 100101, China; Key Laboratory of Organ Regeneration and Reconstruction, Chinese Academy of Sciences, Beijing 100101, China; State Key Laboratory of Stem Cell and Reproductive Biology, Institute of Zoology, University of Chinese Academy of Sciences, Chinese Academy of Sciences, Beijing 100101, China; Institute for Stem Cell and Regeneration, Chinese Academy of Sciences, Beijing 100101, China; Beijing Institute for Stem Cell and Regenerative Medicine, Beijing 100101, China; Key Laboratory of Organ Regeneration and Reconstruction, Chinese Academy of Sciences, Beijing 100101, China; State Key Laboratory of Stem Cell and Reproductive Biology, Institute of Zoology, University of Chinese Academy of Sciences, Chinese Academy of Sciences, Beijing 100101, China; Institute for Stem Cell and Regeneration, Chinese Academy of Sciences, Beijing 100101, China; Beijing Institute for Stem Cell and Regenerative Medicine, Beijing 100101, China; Key Laboratory of Organ Regeneration and Reconstruction, Chinese Academy of Sciences, Beijing 100101, China; State Key Laboratory of Stem Cell and Reproductive Biology, Institute of Zoology, University of Chinese Academy of Sciences, Chinese Academy of Sciences, Beijing 100101, China; Institute for Stem Cell and Regeneration, Chinese Academy of Sciences, Beijing 100101, China; Beijing Institute for Stem Cell and Regenerative Medicine, Beijing 100101, China; Key Laboratory of Organ Regeneration and Reconstruction, Chinese Academy of Sciences, Beijing 100101, China; State Key Laboratory of Stem Cell and Reproductive Biology, Institute of Zoology, University of Chinese Academy of Sciences, Chinese Academy of Sciences, Beijing 100101, China; Institute for Stem Cell and Regeneration, Chinese Academy of Sciences, Beijing 100101, China; Beijing Institute for Stem Cell and Regenerative Medicine, Beijing 100101, China; Key Laboratory of Organ Regeneration and Reconstruction, Chinese Academy of Sciences, Beijing 100101, China; State Key Laboratory of Stem Cell and Reproductive Biology, Institute of Zoology, University of Chinese Academy of Sciences, Chinese Academy of Sciences, Beijing 100101, China; Institute for Stem Cell and Regeneration, Chinese Academy of Sciences, Beijing 100101, China; Beijing Institute for Stem Cell and Regenerative Medicine, Beijing 100101, China; Key Laboratory of Organ Regeneration and Reconstruction, Chinese Academy of Sciences, Beijing 100101, China; State Key Laboratory of Stem Cell and Reproductive Biology, Institute of Zoology, University of Chinese Academy of Sciences, Chinese Academy of Sciences, Beijing 100101, China; Institute for Stem Cell and Regeneration, Chinese Academy of Sciences, Beijing 100101, China; Beijing Institute for Stem Cell and Regenerative Medicine, Beijing 100101, China; Key Laboratory of Organ Regeneration and Reconstruction, Chinese Academy of Sciences, Beijing 100101, China; State Key Laboratory of Stem Cell and Reproductive Biology, Institute of Zoology, University of Chinese Academy of Sciences, Chinese Academy of Sciences, Beijing 100101, China; Institute for Stem Cell and Regeneration, Chinese Academy of Sciences, Beijing 100101, China; Beijing Institute for Stem Cell and Regenerative Medicine, Beijing 100101, China; Key Laboratory of Organ Regeneration and Reconstruction, Chinese Academy of Sciences, Beijing 100101, China; State Key Laboratory of Stem Cell and Reproductive Biology, Institute of Zoology, University of Chinese Academy of Sciences, Chinese Academy of Sciences, Beijing 100101, China; Institute for Stem Cell and Regeneration, Chinese Academy of Sciences, Beijing 100101, China; Beijing Institute for Stem Cell and Regenerative Medicine, Beijing 100101, China; Key Laboratory of Organ Regeneration and Reconstruction, Chinese Academy of Sciences, Beijing 100101, China; State Key Laboratory of Stem Cell and Reproductive Biology, Institute of Zoology, University of Chinese Academy of Sciences, Chinese Academy of Sciences, Beijing 100101, China; Institute for Stem Cell and Regeneration, Chinese Academy of Sciences, Beijing 100101, China; Beijing Institute for Stem Cell and Regenerative Medicine, Beijing 100101, China; Key Laboratory of Organ Regeneration and Reconstruction, Chinese Academy of Sciences, Beijing 100101, China

**Keywords:** single-cell multi-omics, ovarian somatic cells, evolutionary conservation and divergence, theca cells

## Abstract

The ovary plays a crucial role in the reproductive system of female mammals by producing mature oocytes through folliculogenesis. Non-human model organisms are extensively utilized in research on human ovarian biology, thus necessitating the investigation of conservation and divergence in molecular mechanisms across species. In this study, we employed integrative single-cell analysis of transcriptome and chromatin accessibility to identify the evolutionary conservation and divergence patterns of ovaries among humans, monkeys, mice, rats, and rabbits. Our analyses revealed that theca cells exhibited the most significant changes during evolution based on scRNA-seq and scATAC-seq datasets. Furthermore, we discovered common *cis*-regulatory architectures in theca cells across species by conducting joint analyses of scRNA-seq and scATAC-seq datasets. These findings have potential applications in non-human biomedical and genetic research to validate molecular mechanisms found in human organisms. Additionally, our investigation into non-coding genomic regions identified intergenic highly transcribed regions (igHTRs) that may contribute to the evolution of species-specific phenotypic traits. Overall, our study provides valuable insights into understanding the molecular characteristics of adult ovaries while offering new perspectives for studying human ovarian physiology and diseases.

## Introduction

The ovary is an important endocrine and reproductive organ in female mammal, composing of oocytes and ovarian somatic cells [1]. Folliculogenesis is a sophisticated event in the mammalian ovary, that not only requires the support of surrounding somatic cells but also essential hormonal signals and remodeling of ovarian structure [2, 3]. Granulosa cells (GCs), stromal cells (SCs), and theca cells (TCs) are vital for structural support and hormone synthesis. Granulosa cells gradually undergo a transformation from a squamous or cuboid shape to a cubic shape, and this alteration in granulosa cell morphology is closely related to steroidogenesis and cell proliferation in ovary [4–6]. Theca cells surround the granulosa cells, forming an envelope comprising the theca interna housing theca endocrine cells, and the theca externa originating from fibroblast-like cells [7]. Additionally, the theca layer facilitates nutrient transport to granulosa cells, cumulus cells, and oocytes through vascular tissue [8]. Stromal cells expressing BMP4 and BMP7 influence early follicles development [9]. Thus, the coordinated development of these cells is important to folliculogenesis.

The dysfunction of ovarian somatic cells can cause the unbalance of reproductive endocrine function, and then affects oocyte quality and reproductive health [10, 11]. In the past period, researchers have been able to explore the early development of the ovary, cellular composition, ovarian aging, and diseases, as well as other physiological and pathological processes in humans or other model organisms in more detail [12–21], with the advancement of single-cell sequencing technology. For instance, Fan et al. utilized single-cell transcriptome technology to analyze the composition of human ovarian cells [13], and Wang et al. drew a cellular atlas of the ovary and reported transcriptional regulatory characteristics during primordial follicle assembly in mice [20]. Wang et al. unravelled cell type specific mechanisms of ovarian aging and identified new diagnostic markers by non-human primate model [21]. Morris et al. cataloged dynamic changes of ovarian cells during the entire estrous cycle in mice [17], and Lengyel et al. explored the changes in the cellular composition of the normal postmenopausal ovary by single-cell transcriptome and chromatin accessibility [15].

However, current research mostly focuses on a single species and transcriptome sequencing, the conserved and species-specific features in the transcriptomes and chromatin states of adult ovarian somatic cells between human and other model organisms remain an incompletely understood area. For ethical reasons, it is not allowed to use human organisms for biomedical or genetic experiments to study ovary. Thus, it is important to figure out the conservation and divergence of molecular mechanisms across species. Here we generated a single-cell multi-omics map of the ovaries from monkey, mouse, rat and rabbit by 10x Genomics Chromium Single Cell Multiome ATAC + Gene Expression platform, and integrated a reported adult human multi-omics ovarian dataset [14] for revealing the conservation and divergence of ovarian somatic cells across species. Our comprehensive analysis provides new insights into transcriptomic and epigenetic evolutionary process of ovarian somatic cells. Furthermore, *cis*-regulatory architecture of ovarian somatic cells identified by conjoint analyses of scRNA-seq and scATAC-seq datasets figured out overlapping epigenetic regulatory across species. Besides, this work identified intergenic highly transcribed regions (igHTRs) in non-coding genomic regions that might contribute to the evolution of species-specific phenotypic traits. This work is advantageous to better understand molecular characteristics of adult ovary and offer new insights for the study of human ovarian physiology and disease.

## Results

### Cross-species transcriptomic conservation and divergence of ovarian somatic cells

**Figure 1.**
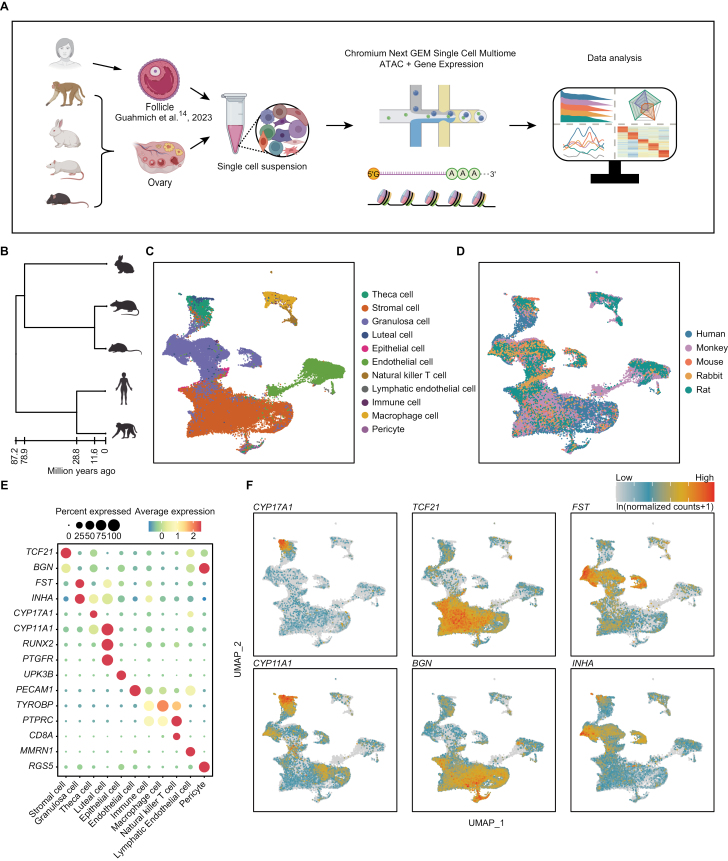
**Single-cell transcriptome profiling across human, monkey, mouse, rat, and rabbit.** (A) Schematic representation of the experimental and bioinformatics workflow. Adapted from BioRender templates. (B) Phylogeny of the five mammals included in this study based on TimeTree5. (C, D) UMAP visualization of the integrated scRNA-seq datasets, colored by cell type (C) and species (D), respectively. (E) Dot plot showing the average expression levels of marker genes in all cell types, respectively. Average expression levels were calculated using natural logarithm-scaled normalized counts. The size of each dot represents the percentage of cell expressing a particular gene. (F) UMAP visualization of the marker genes of theca cells, stromal cells and granulosa cells.

To evaluate the conservation and divergence of ovarian somatic cells in adult mammals, we integrated scRNA-seq data of adult ovaries from five placental mammalian species: one from published data of human (21- to 31-year-old, antral follicles) [14], and four generated data in this study of monkey (6- to 10-year-old), mouse (10-week-old), rat (10- to 22-week-old), and rabbit (20-week-old) (Fig. 1A, 1B and S1B, Table S1). Through strict quality control, a total of 57,408 high-quality single cells were obtained for subsequent analysis (Table S2). The datasets included 13 libraries, with the median gene number more than 1,300 and the median unique molecular identifiers (UMIs) number reaching up to 10,000 (Fig. S1A). The uniform manifold approximation and projection (UMAP) dimension reduction result showed that cells were clustered together according to specific somatic cell types rather than species (Fig. 1C, 1D, and 1E). This result indicated that our data captured the ovarian somatic cell gene expression programming shared by placental mammals, despite their divergent evolution was approximately 90 million years (Fig. 1A and 1B). In accordance with this, the functional genes for specific cell types were expressed in the corresponding cell population (Figs. 1E, 1F and S1C), for example, *CYP17A1, CYP11A1* (coding steroidogenic enzyme) in theca cells [22–24], *TCF21*, *BGN* (former suppresses the expression of *SF1*; later is a paracrine signaling factor) in stromal cells [25, 26], and *FST*, *INHA* (regulating FSH levels) in granulosa cells [27–29] (Figs. 1A, 1B and S1B, Table S1).

**Figure 2.**
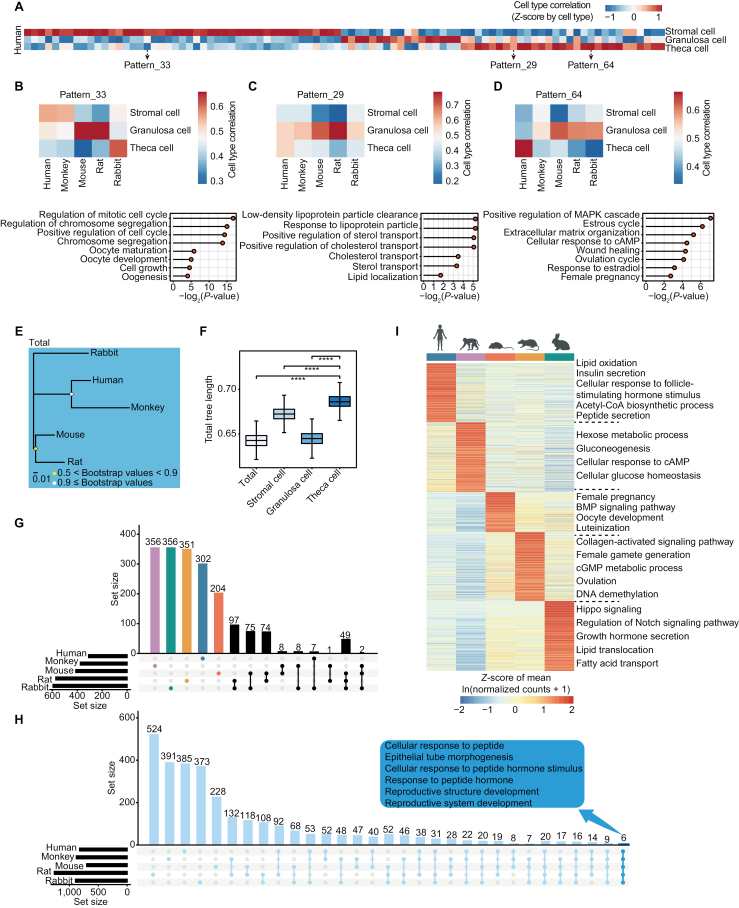
**The comparison of single-cell transcriptomes across species.** (A) Heatmap showing the patterns of gene expression using scCoGAPS algorithm in human cells. The correlation of each pattern to human types was colored. (B–D) Heatmap showing the correlation to three cell types of the pattern 33 (B), 29 (C), and 64 (D) across species (upper); lollipop charts showing the GO terms of genes from corresponding pattern (lower). (E) Phylogeny tree showing the relationships across five species based on pseudo-bulk transcriptomes of whole cells. Bootstrap values were colored. (F) Boxplot showing branch lengths of trees based on pseudo-bulk transcriptomes. *P ≤ 0.05, **P ≤ 0.01, ***P ≤ 0.001, ****P ≤ 0.0001, n.s. denotes not significant (Wilcoxon rank-sum test). (G) UpSet plot showing intersection of species-specifically highly expressed theca cell genes. (H) UpSet plot showing intersection of GO terms of genes identified in Fig. 2G per species. (I) Heatmap showing the scaled average expression levels of species-specifically highly expressed TC genes (detected only once) in each species. The representative GO terms were displayed on the right.

To further explore the transcriptomic features of ovarian somatic cells across species, we used a single-cell coordinated gene activity in pattern sets (scCoGAPS) algorithm to find gene patterns specific to human stromal cells, granulosa cells and theca cells (Fig. 2A). The scCoGAPS is a non-negative matrix factorization (NMF) method to define latent spaces from scRNA-seq dataset, which reveal more about biological systems than marker genes alone [30]. Then, the specific patterns in human somatic cells were projected into the somatic cells of other species (Fig. S2A, S2B, and S2C). Some patterns were conserved across five species, for instance, pattern 52 and pattern 56 (Fig. S2D and S2E). Pattern 52 was involved in Gene Ontology (GO) terms like mesenchymal cell proliferation, Wnt/BMP signaling pathway and response to peptide hormone, and pattern 56 was involved in GO terms like steroid hormone biosynthetic process, such as, estrogen (Fig. S2F and S2G). However, some patterns for theca cells were only identified in one species (Fig. 2B, 2C, and 2D). For example, pattern 33 which was specific in rabbit theca cells was enriched in GO terms like oocyte development and maturation. Pattern 29 related to sterol or cholesterol transport and pattern 64 related to estrous cycle were specific in human theca cells.

To explore the relationship of ovarian cells across species from an evolutionary aspect, we constructed the gene expression trees based on the pseudo-bulk transcriptomes of the whole ovaries (Fig. 2E), stromal cells, granulosa cells and theca cells, respectively (Fig. S2H), which were consistent with the known mammalian phylogeny development (Fig. 1B). The total branch length of trees of somatic cells showed that theca cells had the longest length (Fig. 2F). This indicted that the theca cells obtained more evolutionary expression changes. In ovaries, theca cells play an important role in maintaining follicular structural integrity, facilitating nutrient transport, and synthesizing essential steroid hormones (such as, testosterone and estradiol) [7, 8]. Comparative analysis of theca cell gene expression across species using the Seurat tool ‘FindMarkers’ function identified differentially expressed genes across species in TCs (Fig. 2I and 2G). GO analysis revealed that genes highly expressed in human TCs were related to lipid metabolism and hormone secretion, such as, lipid oxidation and insulin secretion. Insulin has been reported to stimulate the production and secretion of androgens by the ovarian theca cells [31]. Highly expressed genes in monkey TCs were related to carbohydrate metabolism such as hexose metabolic process and gluconeogenesis. Highly expressed genes in mouse TCs were associated with BMP signaling pathway and oocyte development. BMPs can act directly on the theca cell to inhibit androgen production [8]. Female gamete generation related genes were highly expressed in rat TCs. Hippo signaling and Notch signaling pathway related genes were highly expressed in rabbit TCs. Besides, six functions involving the reshaping of reproductive structures and response to peptide hormones (Fig. 2H) were enriched across the five species. These GO terms enriched by the species-specifically highly expressed genes were consistent to reported TCs functions during folliculogenesis. Taken together, although the gene expression patterns of TCs varied across species, the functions of TCs were conservative.

### The epigenetic changes during evolutionary process of ovarian cells across human, monkey, mouse, rat, and rabbit

**Figure 3.**
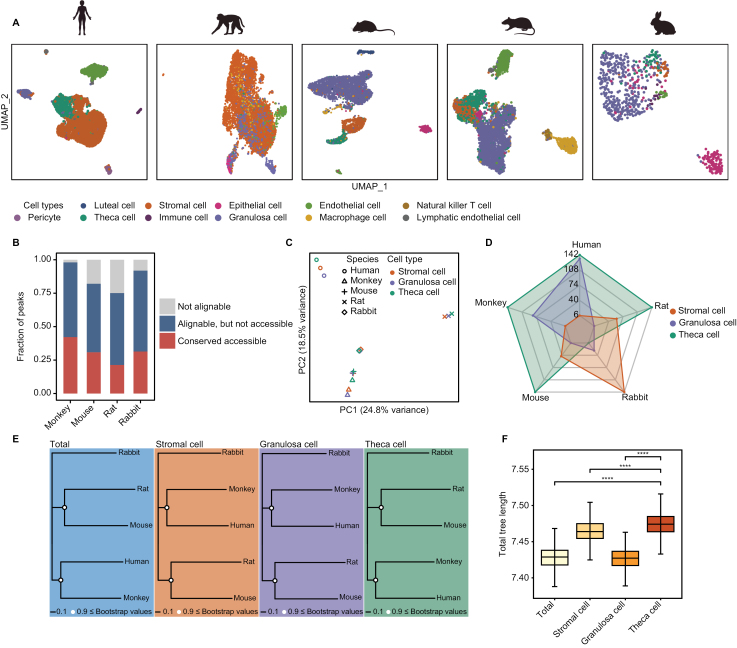
**Comparison of single-cell ATAC-seq datasets across species.** (A) UMAP visualization of the single-cell chromatin accessibility, colored by cell type. (B) Bar plot showing the fraction of three categories of peaks. (C) PCA visualization based on conserved peaks; colored by cell types and shaped by species. (D) Radar chart showing the distance in PC2 among granulosa cells, stromal cells and theca cells, related to Fig. 3C. (E) Phylogeny tree showing the relationships across five species based on the sequences of conserved peaks. Bootstrap values were colored. (F) Boxplot showing branch lengths of trees based on the sequences of conserved peaks. *P ≤ 0.05, **P ≤ 0.01, ***P ≤ 0.001, ****P ≤ 0.0001, n.s. denotes not significant (Wilcoxon rank-sum test).

To investigate the epigenetic changes of evolution in mammalian ovarian somatic cells, we simultaneously performed single-cell sequencing assay for transposase-accessible chromatin (scATAC-seq) of ovarian somatic cells in humans, monkeys, mouses, rats and rabbits (Table S1). After filtering out low-quality nuclei, a total of 23,804 single nuclei with high-quality open chromatin profiles (5,189 for human, 7,263 for monkey, 4,788 for mouse, 5,951 for rat and 613 for rabbit) were obtained from scATAC-seq data. Then, we integrated scRNA-seq and scATAC-seq by identifying shared patterns in the gene activity matrix and gene expression matrix. The cell identities were assigned using cross-modality integration and label transfer (Fig. 3A), and the representative genes exhibited high accessibility levels (Figs. 1F and S3).

Next, we converted monkey, mouse, rat and rabbit peaks coordinates into human genome coordinates using liftOver to address the evolutionary relationships of chromatin accessibility across species. Peaks were categorized into “Not alignable” (non-conserved DNA sequence) and “others” (conserved DNA sequence). “Others” was further divided into “Alignable, but not accessible” (conserved DNA sequence but not open chromatin regions in other species) and “Conserved accessible” (conserved DNA sequence and open chromatin regions in other species) [32]. Consistent with the evolutionary distances of species in the phylogenetic tree, our results revealed that the proportion of “Conserved accessible” regions decreased sequentially in monkey, mouse and rat, ranging from approximately 40% to 20% (Fig. 3B). PCA (principal components analysis) was done based on “Alignable, but not accessible” and “Conserved accessible” peaks, and interestingly, the human, monkey, mouse and rat theca cells were further away from stromal cells and granulosa cells (Fig. 3C and 3D). The phylogenetic trees built by the genetic distance of DNA sequence of conserved peaks among five species from stromal cells, granulosa cells and theca cells documented that theca cells acquired more changes during evolutionary process (Fig. 3E and 3F), which was consistent with the phylogenetic trees built by gene expression (Fig. 2F).

### *Cis*-regulatory architecture of theca cells across species

**Figure 4.**
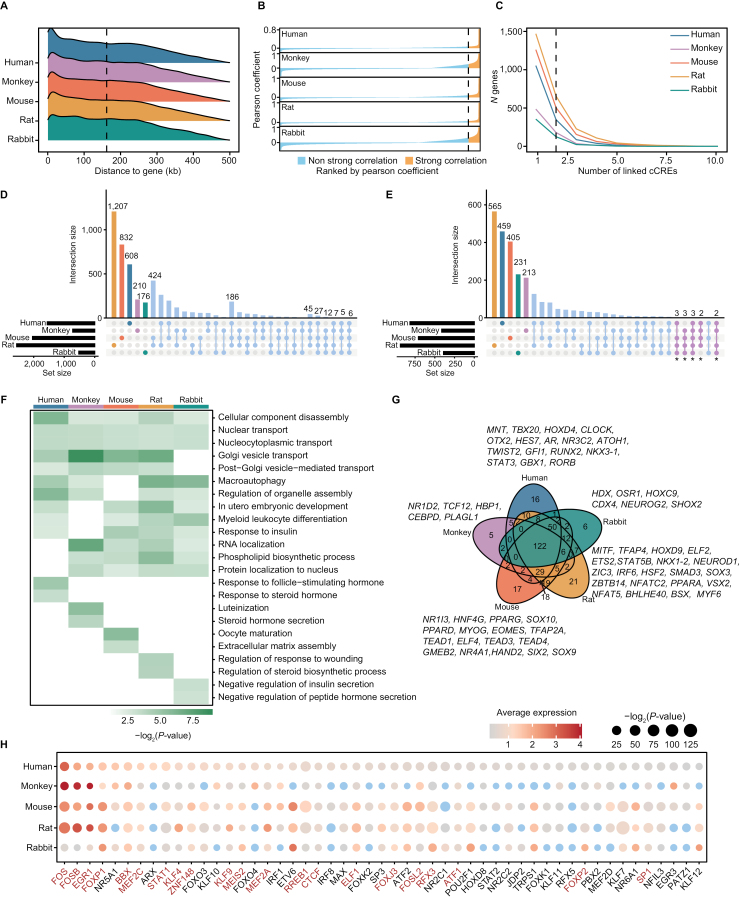
**Analysis of *cis*-regulatory elements in theca cells of different species.** (A) Density plot showing the distributions of distance between cCREs and genes, dash line indicates the median distance. (B) Line plot showing the ordered Pearson’s *r* between chromatin accessibility of cCREs and expression of their target genes, dash line indicates 95th Pearson’s *r*. (C) Line plot showing the number of cCREs linked by genes, dash line indicates *x* = 2. (D) UpSet plot showing the intersection of gl-cCREs genes. (E) UpSet plot showing GO terms of gl-cCREs genes per species, sets marked with asterisk (*) were shown in Fig. 4F. (F) Heatmap showing the conserved and non-conserved GO terms in Fig. 4E. (G) Venn diagram showing the overlaps of motif enrichment by gl-cCREs. (H) Dot plot showing the average expression levels of transcription factors expressing in human theca cells. The size of each dot represents the *P*-value and the color of each dot represents average expression levels (blue indicates no expression, average expression levels ≤ 0.2).

Since both phylogenetic trees based on scRNA-seq and scATAC-seq documented that theca cells changes most among the three ovarian somatic cells during evolution, we further performed cojoint analyses of the two datasets to elucidate the *cis*-regulatory architecture of theca cells across species [33]. First, we identified the candidate *cis*-regulatory elements (cCREs) and their target genes which were obtained from co-accessible peaks annotated as promoter elements. The theca cell cCREs with similar distributions of distance from genes across species (Fig. 4A) and chromatin accessibility of which having a strong positive correlation (Pearson’s *r* > 95th Pearson’s *r*) with target genes (Fig. 4B), were defined as gene-linked cCREs (gl-cCREs) (Table S4). A gene was linked with 1 to 3 cCREs (Fig. 4C), and the majority of gl-cCREs in five species were mapped to intronic regions (Fig. S4A). For all species in this study, we found little overlap between gl-cCREs genes and species-specifically highly expressed theca cell genes identified by scRNA-seq datasets (Figs. 2G, 2I, and S4B). This result may indicate a different role of gl-cCREs genes in theca cell transcriptomic and epigenetic evolution.

Next, we analyzed the function of gl-cCREs genes in each species, and found gl-cCREs genes of different species were partially overlapped and some GO terms were shared (Fig. 4D and 4E). For instance, cellular component disassembly, nucleocytoplasmic transport and nuclear transport were enriched in all the five species (Fig. 4F). Moreover, motif enrichment analysis of gl-cCREs by MEME SEA showed that 122 TFs (transcription factors) were shared across five species (Fig. 4G). 51 of the 122 TFs were expressed (average normalized expression > 0.2) in human and 21 of 51 TFs were conservatively expressed across the five species (Fig. 4H; Table S5). For example, *FOSB* was reported as a hub gene of transcriptional regulatory in monkey ovarian somatic cells and *FOS* affected somatic cell apoptosis and oocyte maturation [21, 34, 35]. Besides, some studies documented that *EGR1* was regulated by FSH and LH, and it mediated the proliferation of theca cells by FGF signaling during folliculogenesis [36, 37]. These results revealed common epigenetic regulatory across species, which could be candidate of biomedical and genetic research contents in non-human organisms to validate findings of human data.

### The evolution of transcriptional activities at non-annotated genome regions of ovaries across species

**Figure 5.**
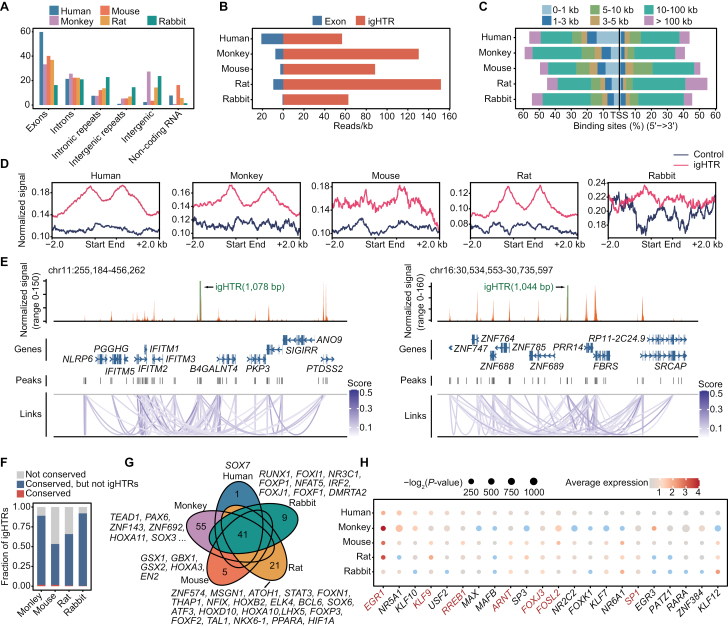
**Analysis of highly transcribed intergenic regions.** (A) Bar plot showing the percentages of annotated genomic elements in theca cell transcriptome alignment regions. (B) Bar plot showing the expression levels (reads per 1 kb) of exons and igHTRs in theca cells. (C) Bar plot showing the distribution of distance between igHTRs and TSS in theca cells. (D) Line plots showing the chromatin accessibility of igHTRs, and their 2 kb upstream and downstream. As control, random genomic regions were selected in corresponding species. (E) Cicero co-accessibility at selected igHTRs (± 100 kb) in human theca cells, colored by the score of co-accessibility. Arcs connecting peaks represent co-accessibility. (F) Bar plot showing the fraction of three categories of igHTRs. (G) Venn diagram showing the overlaps of motif enrichment in igHTRs. (H) Dot plot showing the average expression levels of transcription factors expressing in human theca cells. The size of each dot represents the *P*-value and the color of each dot represents average expression levels (blue indicates no expression, average expression levels ≤ 0.2).

To assess if other genomic regions (non-coding regions) have transcriptional activity and contribute to evolution of species-specific phenotypic traits, we aggregated our scRNA-seq reads as pseudo-bulk base on species and cell types. Generally, more than 50% of the reads were mapped to exons and introns regions (included repeats), and the remaining reads were mapped within non-coding and intergenic regions (Fig. S5A). Such distribution of transcriptome reads was common in the five species, but the percentage of genomic regions varied among species. Taken theca cells as example, nearly 60% of human reads were mapped to exons regions, more than 20% of monkey and rabbit reads were mapped to intergenic regions without repeats, and 14.1% of mouse reads were annotated to non-coding RNA regions (Fig. 5A). We also explored the distribution of reads in independent samples of human and monkey, and the similar pattern was obtained (Fig. S5B). Without taking into account of differences in the quality of genome annotation among species, this might indicate the evolution of different genomic regions.

To characterize intergenic regions (without repeats) in theca cells in depth, we merged the regions and calculated the expression levels of intergenic regions. Notably, the expression levels of such regions greatly exceed the expression levels of exons for each species and we defined these regions as intergenic highly transcribed regions (igHTRs) [38] (Fig. 5B; Table S6). The length of igHTRs was mainly distributed between 300 and 600 bp (Fig. S5C), which was slightly shorter in rabbits and the great majority of reads were 10–100 kb away from the transcription start site (TSS) (Fig. 5C). In addition, igHTRs were of high chromatin accessibility (Fig. 5D) and some igHTRs were co-accessible with TSS in theca cells of primates and non-primates (Fig. 5E). To evaluate conservativeness of igHTRs across species from the aspect of chromatin accessibility, we converted monkey, mouse, rat and rabbit igHTRs coordinates into human genome coordinates by liftOver. The igHTRs were firstly classed into “Not conserved” (non-conserved DNA sequence) and “others” (conserved DNA sequence). “Others” was further divided into “Conserved, but not igHTRs” (conserved DNA sequence but not igHTRs in human) and “Conserved” (conserved DNA sequence and overlapped with human igHTRs). These results showed that the other species igHTRs shared conserved DNA sequence with human (Fig. 5F), but their chromatin accessibility differed from human igHTRs (Fig. S5D).

From another aspect to evaluate the conservativeness of igHTRs, the TF enrichment was performed using igHTRs from five species and 41 TFs were enriched in five species (Fig. 5G). Based on scRNA-seq data, 22 of the 41 TFs were expressed in human theca cells and 7 of 22 TFs were conserved across five species (Fig. 5H), for example, *FOSL2*, which is involved in the regulation of *CYP11A1* and the later encodes the enzyme P450scc related to the production of pregnenolone [39, 40], and *KLF9*, a gene that is reported to regulate the activity of progesterone receptor [41]. These results indicated that the non-annotated genome regions can contribute to the evolution and conservativeness of TCs across the five species.

## Discussion

To date, several non-human model organisms were employed to explore molecular mechanism found in human ovary using biomedical and genetic approaches. However, the credibility of conclusions made by non-human organisms needs to be carefully evaluated due to differences between species and it is necessary to study the evolutionary conservation and divergence of ovary across species. Integration of single-cell multi-omics has been used to catalog cell states and activities in various species [42] and this can improve our knowledge of the transcriptional regulatory program in adult ovarian cells. There is a lack of comprehensive analysis of ovarian cells across species from a molecular point of view.

**Figure 6.**
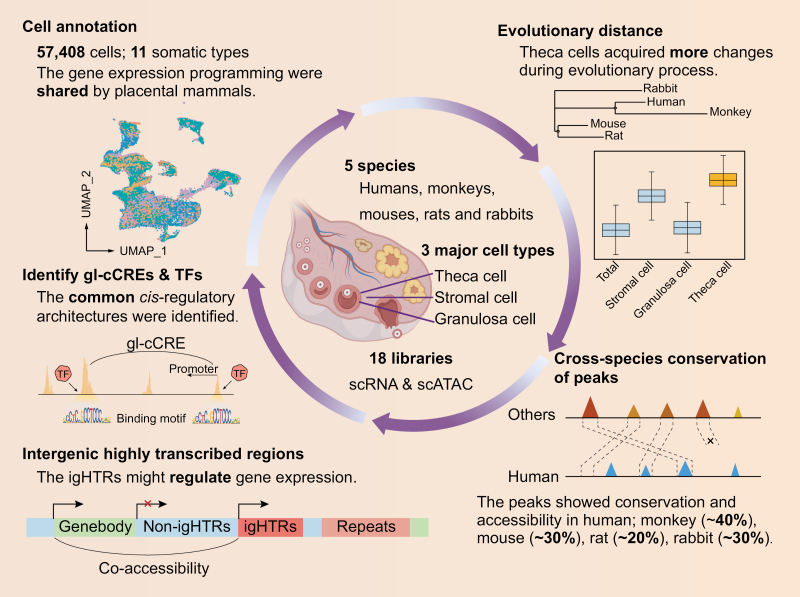
**Graphical summary.** The conservation and divergence of ovarian somatic cells were characterized by transcriptome and chromatin accessibility across rodents, monkeys, and humans.

The integrative comparison of the scRNA-seq and scATAC-seq between human and other model organisms in our study uncovered significant evolutionary changes across species, including gene expression patterns, open chromatin regions, gene-linked regulatory elements, and non-coding genomic regions (Fig. 6). Moreover, the evolution processing of ovarian somatic cells was varied, with the theca cells having the most evolutionary changes, both in gene expression and chromatin accessibility (Figs. 2F and 3F), while the granulosa cells being the most conservative. These results highlighted the importance of evaluating the evolutionary conservation and divergence when selecting a model system. Thus, choosing common *cis*-regulatory architecture across human and other species as candidate biomedical and genetic research contents in non-human organisms would draw more convincing conclusion for the study of human ovarian physiology and disease. Our conjoint analyses of scRNA-seq and scATAC-seq datasets revealed such common molecular characteristics even in the most different cell types among species.

In summary, this is a comparative frontier single-cell multi-omics analysis of ovaries across species. Our work deepens the understanding of the transcriptome and epigenetic features of ovarian somatic cells in adult, uncovers the evolutionary conservation and divergence of ovary across species, and provides new insights for the study of human ovarian physiology and disease.

## Research limitations

Combining other omics, such as methylome and proteome will be helpful to find more conservative regulatory architecture across species. In addition, higher quality of genome annotation is needed for cross-species analysis.

## Methods

### Research ethics

The datasets of human ovaries were downloaded from the GEO database (GSE192722). And the experiments of cynomolgus monkeys were conducted according to the Principles for the Ethical Treatment of Non-human Primates and were approved in advance by the Institutional Animal Care and Use Committee of the Institute of Zoology (IOZ-IACUC-2021-178). The experiments of mouse, rats and rabbits were conducted according to the standard operating procedure of the Institute of Zoology (IOZ-IACUC-2020-107).

All applicable institutional and national guidelines for the care and use of animals were followed in this study.

### Ovary collection and single-cell dissociation

Single-cell isolation was performed based on a protocol described previously with modification [43]. In brief, animals were killed following experimental animal welfare and ovaries were collected for single-cell dissociation. Then, ovaries from same species were transferred into a 1.5 mL tube with 400 μL M2 medium (Sigma–Aldrich, M7167) and 50 μL collagenase IV (1 mg/mL, Gibco, 17104-019), and incubates at 37°C under 1,000 rpm for 15–20 minutes. Then adding 200 μL 0.05% TrypLE Express Enzyme (Gibco, 12604021) into tube after removing the supernatant and incubates at 37°C under 1,000 rpm for 20–30 minutes. Next, 400 μL M2 medium was added to stop digestion and pipette for 50–100 times to dissociate the ovary into cell suspension. Then spin down the cells on Mini-spin for 30 s and removing the supernatant, 400 μL M2 medium was added to resuspend the cells (repeat once). Finally, removing the supernatant, cells were resuspended with 1× DPBS and 0.04% BSA. These cells were counted using a cell counter and then used for single-cell sequencing.

### Hematoxylin-eosin staining

Fresh ovaries were fixed, dehydrated, and then the tissues were embedded in paraffin. Next, the embedded ovaries were cut into sections using a rotary microtome (Leica, HistoCore BIOCUT). After deparaffinizing, the sections were processed for hematoxylin-eosin staining. Finally, the tissue sections were dried in fume hood for at least 2 days before imaging using slide scanner (Leica, Aperio VERSA 8).

### Immunofluorescence staining

Fresh ovarian tissues were fixed, dehydrated, and embedded in OCT medium prepared for sectioning using a cryostat microtome (Leica, CM1860). Subsequently, the sections were permeabilized and blocked in turn, followed by incubating with primary antibodies (Abcam, ab134910, 1:100; Santa Cruz, sc-374244, 1:100). Samples were further incubated with secondary antibodies (Thermo Fisher, A-21429, 1:500). Finally, samples were stained using DAPI and imaged under an upright fluorescence microscope (Nikon, Ci-L Plus).

### Single-cell library construction and sequencing

For the construction of scRNA-seq or scATAC-seq libraries, 10x Genomics Chromium Next GEM Single Cell Multiome ATAC + Gene Expression Reagent Bundle were used according to the manufacturer’s instructions. Sequencing was run on Illumina NovaSeq 6000 and roughly 30–40 million reads were obtained per library.

### Genomes and annotations

For human and mouse, reference genomes were obtained from 10x Genomics: human (GRCh38-2020-A-2.0.0), mouse (mm10-2020-A-2.0.0). And for other species, genomes and corresponding annotations were downloaded the from Ensembl: cynomolgus monkey (macFas5), rabbit (OryCun2.0), rat (mRatBN7.2) and then, reference genomes were generated as instructed by Cell Ranger ARC (v2.0, 10x Genomics).

### Alignment of sequencing reads and low-quality cells removal

For sequencing data generated by 10x Genomics platform, raw sequencing reads were aligned to corresponding reference, the count, peak and fragment matrices was generated by Cell Ranger ARC (v2.0, 10x Genomics). For each sample independently, cells were marked low quality were filtered and quality control standards were provided in Table S1.

### Cell type assignment

For single-cell RNA sequencing data, first, Seurat [44] object was created and normalization was performed for each species, respectively. And the high variable genes (HVGs) were identified using the *FindVariableFeatures* with default parameters. The batch correction was then performed by *FindIntegrationAnchors* and *IntegrateData* in monkey data. Next, uniform manifold approximation and projection (UMAP) was performed by *RunUMAP*, followed the cluster identification was done with *FindNeighbors* and *FindClusters*. Subsequently, the cell populations were identified based on the enriched expression of selected markers in each seurat object.

For single-cell ATAC sequencing data, cell type labels were transferred to the scATAC-seq data, based on the same barcodes between scRNA-seq and scATAC-seq data in human, mouse and rat database [45]. Besides, for monkey and rabbit database, TF-IDF matrix from the count matrix was computed by *RunTFIDF* function in Signac. Then, features were selected by using *FindTopFeatures* and the two-dimension visualization were generated by using *RunUMAP*. Cell types were transferred based on single-cell RNA sequencing data by using *FindTransferAnchors* and *TransferData*.

### Integration of single-cell RNA sequencing data

To integrate single-cell RNA sequencing data from different species, 1:1 orthologue genes were obtained by R package biomaRt. Then the previous Seurat objects were filtered by orthologue genes set. Next, for correcting batch effect, the Seurat anchoring approach was applied by *FindIntegrationAnchors* and *IntegrateData* functions with 30 principal components to integrate all datasets together into a single Seurat object. For integrated Seurat object, the two-dimension visualization was done followed as recommended in Seurat.

### Identification of differentially expressed genes and GO enrichment analysis

The differentially expressed genes were identified by using the *FindAllMarkers* or *FindMarkers* functions in Seurat. All GO enrichment analysis was performed by using the R package clusterProfiler [46] and the annotation R package org.Hs.eg.db.

### Peak calling and co-accessible peaks identifying

For peak calling by MACS2 based on cell type, the Seurat objects of scATAC-seq were firstly converted ArchR object based on genome annotations [47, 48]. And then, the pseudo-bulk replicates and reproducible peak sets were generated using the *addGroupCoverages* and *addReproduciblePeakSet*. Next, the peak-to-peak links were identified by using the R package Cicero.

### Transcriptome composition and intergenic highly transcribed regions analysis

The PCR or optical duplicated reads in aligned bam files were filtered by using samtools with -F 1024 refer to previous studies [38]. The filtered bam files were split into individual files based on cell barcodes and bedtools was used to obtained information about alignments of specific genomic regions. For define the intergenic highly transcribed regions (igHTRs), two parameters, the spacing between two neighboring reads less than 150 bp and the number of mapped sequences more 10 was used.

### Motif enrichment within accessible peaks

The SEA tool (simple enrichment analysis) (v5.5.5) was used to identify enriched transcription factor binding motifs within accessible peaks. For each species, the genome regions were inputted into the SEA tool with the motif database (vertebrates (*in vivo* and *in silico*)) and the significantly enriched motifs were identified (*P*-value less than 0.05).

### Statistical analysis

Statistical parameters were reported in corresponding figure legends and all statistical analyses were done in R (v4.1.2).

## Supplementary Material

lnae028_suppl_Supplementary_Figure_S1

lnae028_suppl_Supplementary_Figure_S2

lnae028_suppl_Supplementary_Figure_S3

lnae028_suppl_Supplementary_Figure_S4

lnae028_suppl_Supplementary_Figure_S5

lnae028_suppl_Supplementary_Tables_S1-S6

lnae028_suppl_Supplementary_Figure_Legends

## Data Availability

All raw data of single-cell sequencing have been deposited in the Genome Sequence Archive (GSA: CRA015823). The scRNA-seq and scATAC-seq data of human antral follicles were downloaded from the GEO database (GSE192722).
