# Supplementary figures and images for: The evolution of ovarian somatic cells characterized by transcriptome and chromatin accessibility across rodents, monkeys, and humans
Source: Life Med. 2024 Jul 31;3(5):lnae028. doi: 10.1093/lifemedi/lnae028 (PMC11749874; doi:10.1093/lifemedi/lnae028)

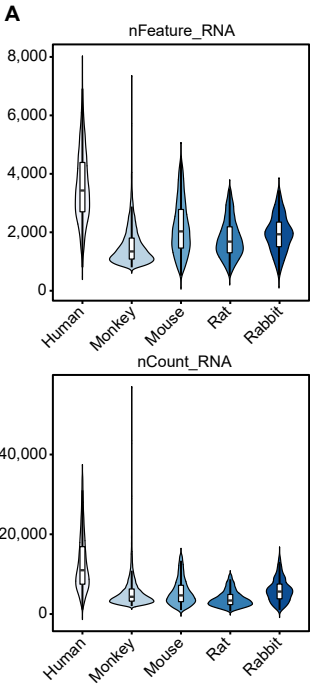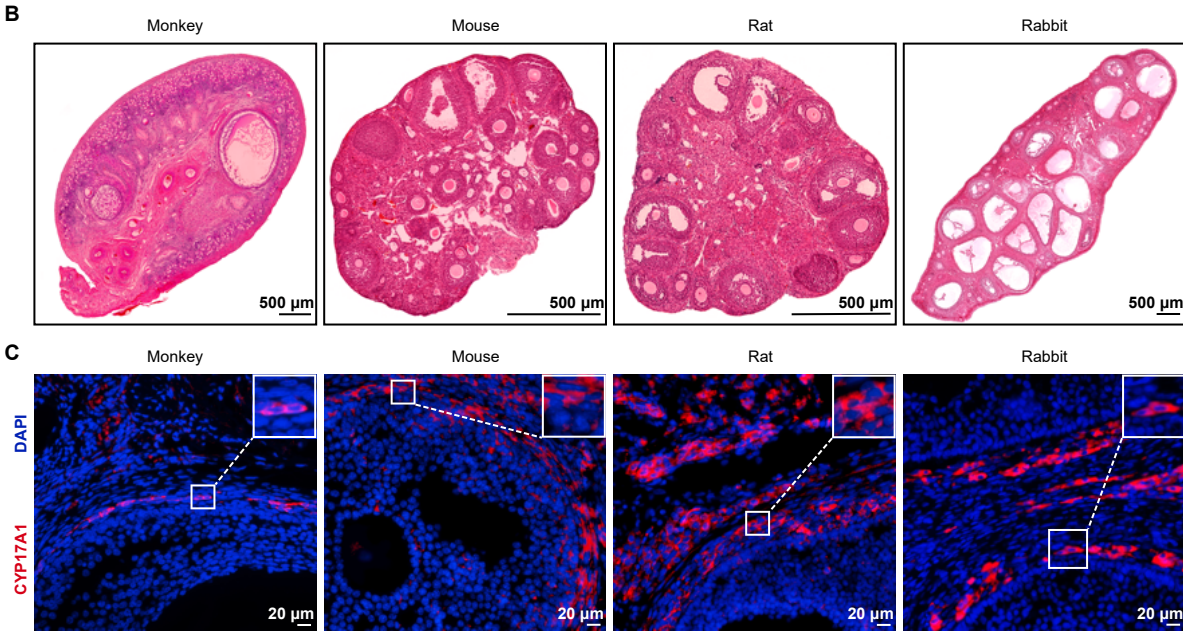

Supplement: lnae028_suppl_Supplementary_Figure_S1 [file lnae028_suppl_Supplementary_Figure_S1.pdf]

Figure S3

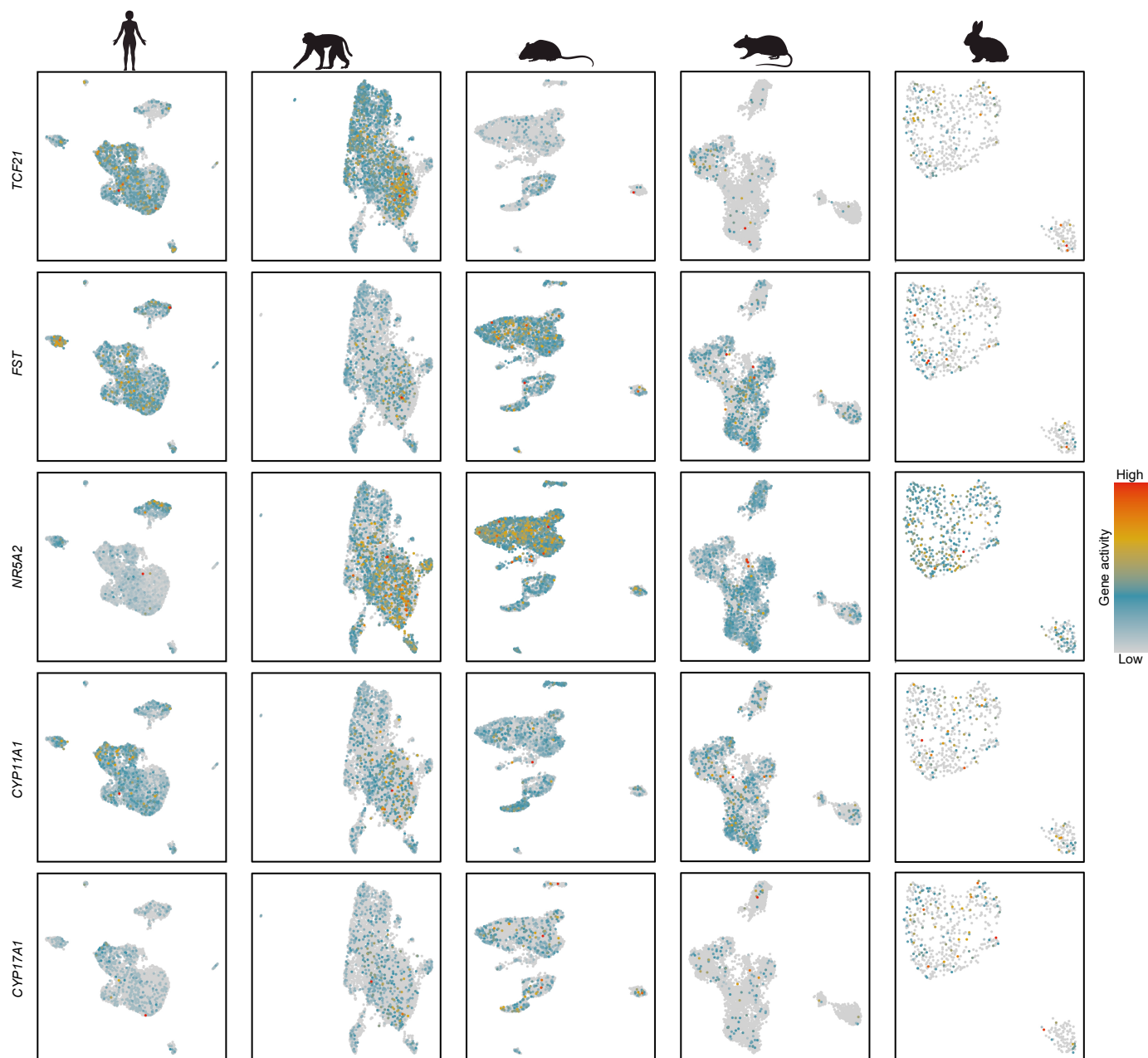

Supplement: lnae028_suppl_Supplementary_Figure_S3 [file lnae028_suppl_Supplementary_Figure_S3.pdf]

Figure S4

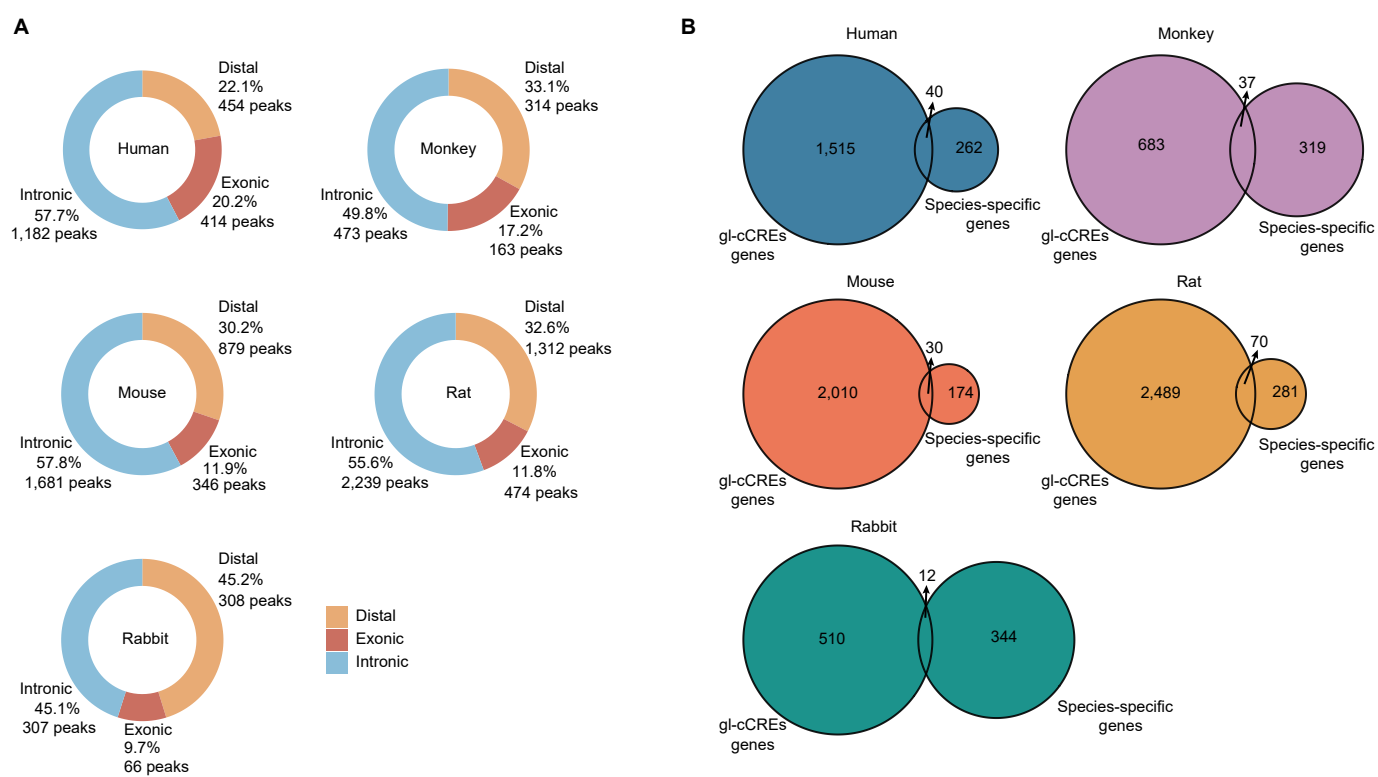

Supplement: lnae028_suppl_Supplementary_Figure_S4 [file lnae028_suppl_Supplementary_Figure_S4.pdf]

Figure S5

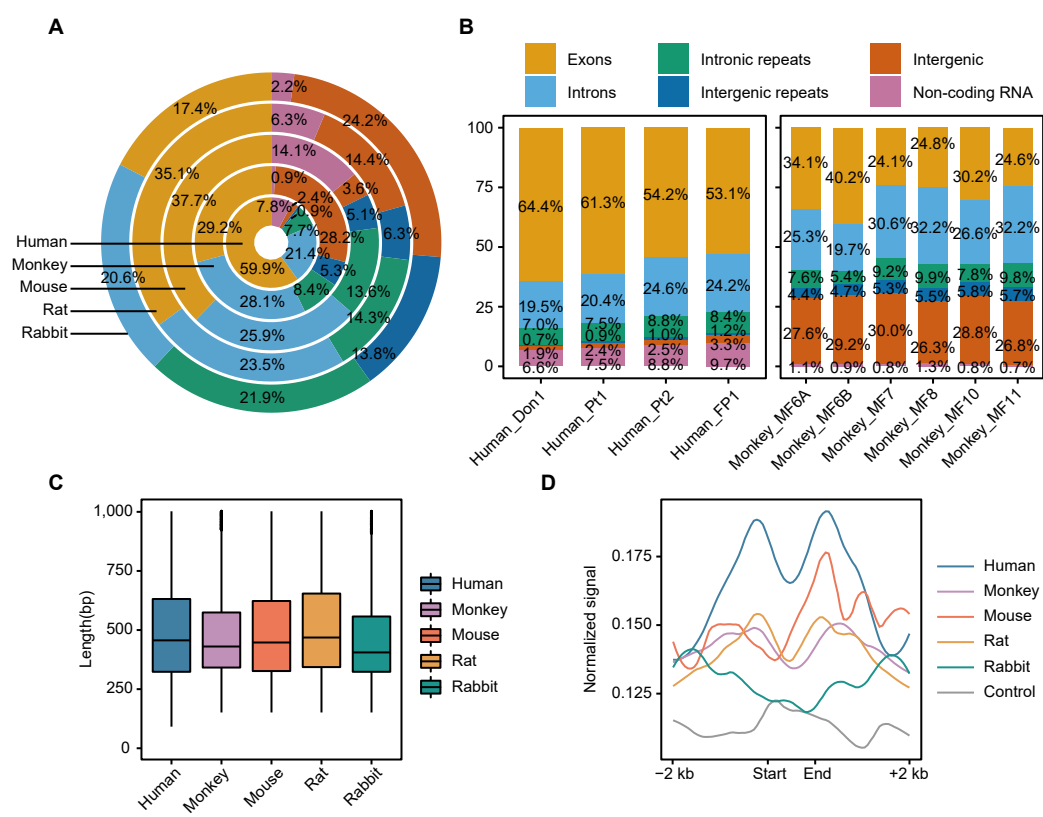

Supplement: lnae028_suppl_Supplementary_Figure_S5 [file lnae028_suppl_Supplementary_Figure_S5.pdf]
