# Supplementary material for: The evolution of ovarian somatic cells characterized by transcriptome and chromatin accessibility across rodents, monkeys, and humans
Source: Life Med. 2024 Jul 31;3(5):lnae028. doi: 10.1093/lifemedi/lnae028 (PMC11749874; doi:10.1093/lifemedi/lnae028)
Supplement: lnae028_suppl_Supplementary_Figure_S2 [file lnae028_suppl_Supplementary_Figure_S2.pdf]

Figure S2

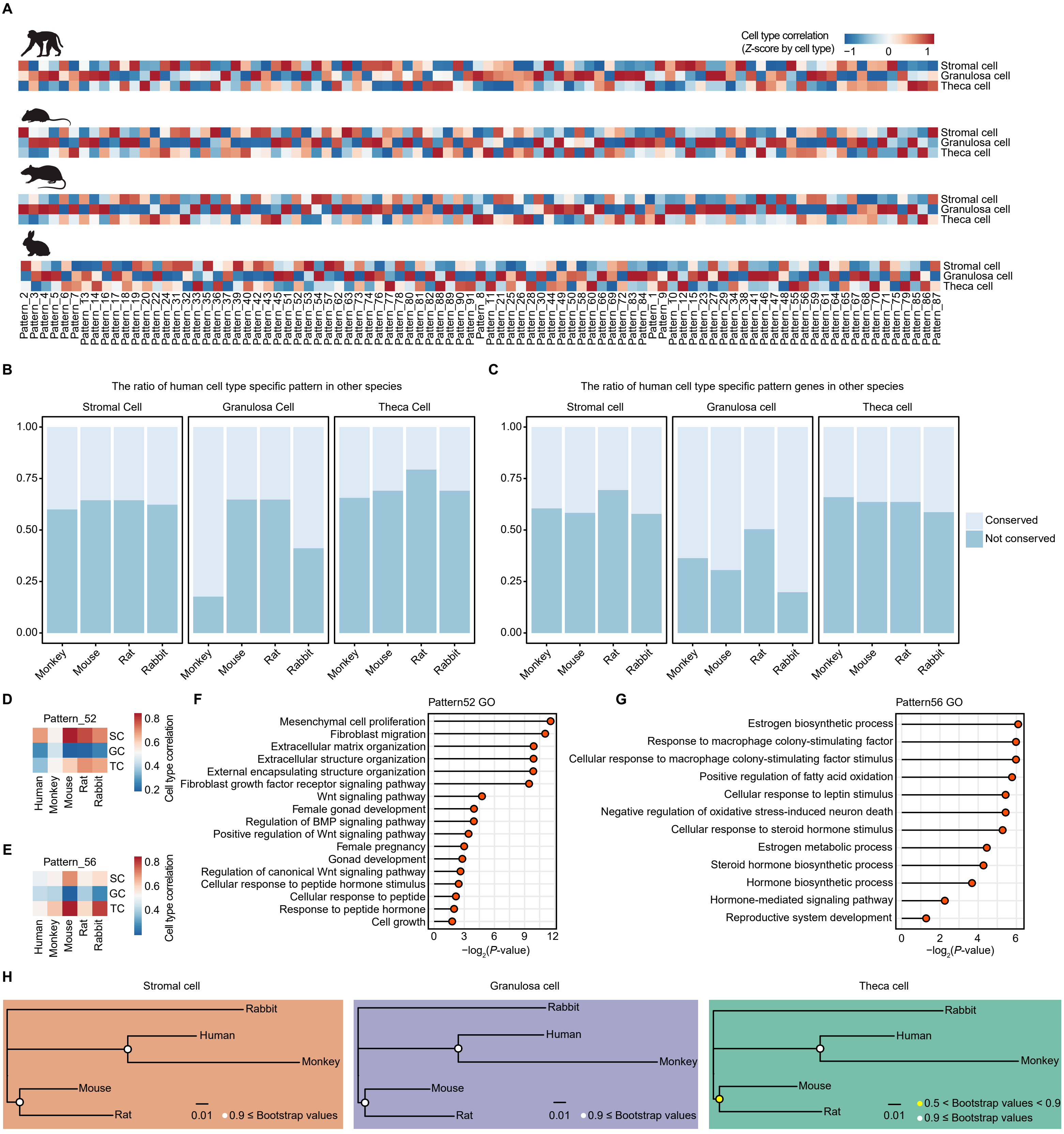

A
Cell type correlation
(Z-score by cell type)
−1
0
1
Stromal cell
Granulosa cell
Theca cell
B
The ratio of human cell type specific pattern in other species
Stromal Cell
Granulosa Cell
Theca Cell
1.00
0.75
0.50
0.25
0.00
Monkey
Mouse
Rat
Rabbit
C
The ratio of human cell type specific pattern genes in other species
Stromal cell
Granulosa cell
Theca cell
Conserved
Not conserved
D
Pattern_52
SC
GC
TC
Human
Monkey
Mouse
Rat
Rabbit
Cell type correlation
E
Pattern_56
F
Pattern52 GO
Mesenchymal cell proliferation
Fibroblast migration
Extracellular matrix organization
Extracellular structure organization
External encapsulating structure organization
Fibroblast growth factor receptor signaling pathway
Wnt signaling pathway
Female gonad development
Regulation of BMP signaling pathway
Positive regulation of Wnt signaling pathway
Female pregnancy
Gonad development
Regulation of canonical Wnt signaling pathway
Cellular response to peptide hormone stimulus
Cellular response to peptide
Response to peptide hormone
Cell growth
−log2(P-value)
G
Pattern56 GO
Estrogen biosynthetic process
Response to macrophage colony-stimulating factor
Cellular response to macrophage colony-stimulating factor stimulus
Positive regulation of fatty acid oxidation
Cellular response to leptin stimulus
Negative regulation of oxidative stress-induced neuron death
Cellular response to steroid hormone stimulus
Estrogen metabolic process
Steroid hormone biosynthetic process
Hormone biosynthetic process
Hormone-mediated signaling pathway
Reproductive system development
H
Stromal cell
Granulosa cell
Theca cell
Rabbit
Human
Monkey
Mouse
Rat
0.01
0.9 ≤ Bootstrap values
0.5 < Bootstrap values < 0.9
