# Supplementary material for: The evolution of ovarian somatic cells characterized by transcriptome and chromatin accessibility across rodents, monkeys, and humans
Source: Life Med. 2024 Jul 31;3(5):lnae028. doi: 10.1093/lifemedi/lnae028 (PMC11749874; doi:10.1093/lifemedi/lnae028)
Supplement: lnae028_suppl_Supplementary_Figure_Legends [file lnae028_suppl_Supplementary_Figure_Legends.docx]

**The evolution of ovarian somatic cells characterized by transcriptome and chromatin accessibility across rodents, monkeys and humans**

Qiancheng Zhang^1,2,3,4,5^, Fengyuan Sun^1,2,3,4,5^, Ruifeng Zhang^1,2,3,4,5^, Donghong Zhao^1,2,3,4,5^, Ran Zhu^1,2,3,4^, Xin Cheng^1,2,3,4^, Xin Long^1,2,3,4^, Xinling Hou^1,2,3,4^, Rui Yan^1,2,3,4^, Yu Cao^1,2,3,4^, Fan Guo^1,2,3,4,#^, Long Yan^1,2,3,4,*^, Yuqiong Hu^1,2,3,4,*^

^1^State Key Laboratory of Stem Cell and Reproductive Biology, Institute of Zoology, University of Chinese Academy of Sciences, Chinese Academy of Sciences, Beijing 100101, China.

^2^Institute for Stem Cell and Regeneration, Chinese Academy of Sciences, Beijing 100101, China.

^3^Beijing Institute for Stem Cell and Regenerative Medicine, Beijing 100101, China.

^4^Key Laboratory of Organ Regeneration and Reconstruction, Chinese Academy of Sciences, Beijing 100101, China.

^5^These authors contributed equally.

^#^Senior author

*Correspondence: yqhu@ioz.ac.cn (Y.H.), yanlong@ioz.ac.cn (L.Y.)

**Figure S1. Quality control of single-cell RNA sequencing.**

(A) Violin plot showing the number of detected genes (left), and unique molecular identifiers (UMIs) (right) per cell.

(B) Representative images of ovarian morphology. Scale bar, 500 μm.

(C) Immunofluorescence staining of CYP17A1. Nuclei were counterstained with DAPI. Scale bar, 20 μm.

**Figure S2. Comparative analysis of gene expression patterns of ovarian somatic cells in five species.**

(A) Heatmap showing the patterns of gene expression using scCoGAPS algorithm. The correlation of each pattern to cell types was colored.

(B) Bar plots showing the ratio of conserved and non-conserved pattern in Fig. 2A in four species.

(C) Bar plots showing the ratio of genes from conserved and non-conserved pattern in Fig. S2B in four species.

(D, E) Heatmap showing the correlation to three cell types of the pattern 52 (D), and 56 (E) across species. SC, stromal cell; GC, granulosa cell; TC, theca cell.

(F, G) Lollipop charts showing the GO terms of genes from pattern 52 (F) and 56 (G).

(H) Phylogeny trees showing the relationships across five species based on pseudo-bulk transcriptomes. Bootstrap values were colored.

**Figure S3. The chromatin accessibility of representative marker genes of granulosa cells, stromal cells and theca cells per species.**

UMAP visualization of marker genes, colored by normalized gene activity.

**Figure S4. The features of gl-cCREs and their target genes.**

(A) Donut charts showing the proportion of gl-cCREs that mapped to different genomic regions.

(B) Venn diagrams showing the overlaps between the gl-cCREs genes and theca cells specific genes in Fig. 2I.

**Figure S5. The distribution of transcriptome alignment regions and the length of igHTRs.**

(A) Donut chart showing the percentages of annotated genomic elements in transcriptome alignment regions.

(B) Bar plots showing the percentages of annotated genomic elements in transcriptome alignment regions, split by sample.

(C) Boxplot showing the length of igHTRs.

(D) Line plots showing the chromatin accessibility of human igHTRs and other species igHTRs that could be converted by liftOver, and their 2 kb upstream and downstream in human. As control, random genomic regions were selected in human.
